# Predicting the involvement of polyQ- and polyA in protein-protein interactions by their amino acid context

**DOI:** 10.1016/j.heliyon.2024.e37861

**Published:** 2024-09-14

**Authors:** Pablo Mier, Miguel A. Andrade-Navarro

**Affiliations:** Institute of Organismic and Molecular Evolution, Faculty of Biology, Johannes Gutenberg University Mainz, Hans-Dieter-Hüsch-Weg 15, 55128 Mainz, Germany

**Keywords:** Homorepeat, Polyglutamine, Polyalanine, Protein-protein interaction, Machine learning

## Abstract

Homorepeats, specifically polyglutamine (polyQ) and polyalanine (polyA), are often implicated in protein-protein interactions (PPIs). So far, a method to predict the participation of homorepeats in protein interactions is lacking. We propose a machine learning approach to identify PPI-involved polyQ and polyA regions within the human proteome based on known interacting regions. Using the dataset of human homorepeats, we identified 157 polyQ and 745 polyA regions potentially involved in PPIs. Machine learning models, trained on amino acid context and homorepeat length, demonstrated high precision (0.90–0.98) but variable recall (0.42–0.85). Random forest outperformed other models (AUC polyQ = 0.686, AUC polyA = 0.732) using the positions surrounding the homorepeat −10 to +10. Integrating paralog information marginally improved predictions but was excluded for model simplicity. Further optimization revealed that for polyQ, using amino acid surrounding positions from −6 to +6 increased AUC to 0.715. For polyA, no improvement was found. Incorporating coiled coil overlap information enhanced polyA predictions (AUC = 0.745) but not polyQ. Finally, we applied these models to predict PPI involvement across all polyQ and polyA regions, identifying potential interactions. Case studies illustrated the method's predictive capacity, highlighting known interacting regions with high scores and elucidating potential false negatives.

## Highlights

• We predict PPI-involved polyQ and polyA regions within the human proteome.
• Model optimization using surrounding amino acid positions improved predictions.
• Optimized models use amino acid positions −6 to +6 for polyQ, −10 to +10 for polyA.

## 1 Introduction

Protein homorepeats, characterized by consecutive repetitions of the same amino acid within a protein sequence, are prevalent in eukaryotic proteomes [1]. These repetitive elements play diverse roles in protein structure, function, and interactions. Homorepeats are often found in regions known as intrinsically disordered regions (IDRs), where they can adopt different conformations and mediate protein-protein interactions (PPIs) [2]. Among the best-studied homorepeats are polyglutamine (polyQ) and polyalanine (polyA) tracts.

PolyQ tracts, consisting of consecutive glutamine residues, have been extensively studied in the context of PPIs, where they often mediate the assembly of large protein complexes [3]. PolyQ regions are associated with neurodegenerative diseases, such as Huntington's disease (HD) and several spinocerebellar ataxias (SCAs) [4]. The expansion of polyQ tracts beyond a critical threshold leads to protein misfolding, aggregation, and neuronal toxicity, ultimately contributing to disease pathogenesis [5].

Similarly, polyA homorepeats have been implicated in mediating PPIs and modulating protein function and stability [6,7]. For instance, polyA tracts within transcription factors have been shown to influence protein-DNA interactions and transcriptional activity [8]. Moreover, polyA-containing proteins can form amyloid-like fibrils and aggregates under certain conditions, similarly to the pathological aggregation observed in polyQ-associated neurodegenerative diseases. These aggregates can sequester essential cellular components, disrupt protein homeostasis, and impair cellular functions, highlighting the significance of polyA in protein interactions and cellular physiology. Not surprisingly, polyA regions have also been associated with neurodegenerative diseases [9].

While polyQ and polyA have been acknowledged for their functional involvement in protein-protein interactions, it is worth noting that most likely not all of these regions actively engage in PPIs. Traditionally, the association between these homorepeats and their functional significance has been established subsequent to characterizing an interaction. So even though the homorepeats themselves can be defined directly from the amino acid sequence using tools such as polyX2 [10], no work has been done to predict their possible functional involvement.

Previous research on using machine learning to predict protein functions from amino acid sequences has achieved notable progress. Early approaches utilized simple classifiers like k-nearest neighbors and decision trees, focusing on sequence motifs and primary structure features [11]. More sophisticated methods, including support vector machines, random forests, neural networks, language models, and graph models have since been employed to capture complex relationships within the data [[12], [13], [14]].

We hypothesize that leveraging existing knowledge of specific homorepeats annotated as interacting regions could enable a machine learning approach to discern which polyQ and polyA regions, among the comprehensive set found in the human proteome, are implicated in PPIs. We compared various machine learning algorithms, particularly highlighting the effectiveness of random forest, to predict PPIs involving polyQ and polyA regions in the human proteome based on amino acid context and homorepeat length. Despite exploring the use of paralogy and coiled coil information, the models showed that homorepeat length and specific surrounding amino acids were key predictors, with polyQ predictions improving with shorter contexts and polyA requiring a wider sequence environment.

## 2 Methods

### 2.1 Data preparation

The complete human reference proteome was downloaded from UniProtKB release 2023_04 (20,596 proteins) [15]. The positional sequence features for the complete proteome, such as coiled coil regions, were also retrieved from the same database and release. We calculated the polyQ and polyA regions in the human proteome with polyX2 [10] and a threshold of 4/6 (a minimum of four glutamines or alanines in a sliding window of six amino acids). Per polyQ and polyA we obtained the regions comprising the ten previous amino acids (positions −10 to −1) and the ten following amino acids (positions +1 to +10). We obtained 2085 polyQ, 7264 polyA, and their respective amino acid contexts following this procedure.

We retrieved a set of 124,923 positionally annotated protein-protein interactions (PPIs) from Interactome3D release 2020_05 [16]. This database gathers structural annotations of protein-protein interaction networks obtained directly from experimentally solved structures of protein complexes deposited in the PDB database [17]. Those PPIs were filtered to 33,800 unique protein regions in which an interaction to a different protein is annotated.

Paralogy information was downloaded from Ensembl release 111 [18], using human dataset GRCh38.p14, for a total of 263,275 paralogous protein pairs.

### 2.2 Tuning the machine learning models

We transformed the context positions of the polyQ and polyA regions, independently, into a table to use in the machine learning procedure. It contains all context positions from −10 to +10 in columns (machine learning variables), and all homorepeats in rows; the cells of the table are the amino acids, with up to 21 possible values: 20 proteinogenic amino acids plus the symbol “-” for a non-existent amino acid, if the homorepeat is separated by less than 10 amino acids from the N- or C-terminal end of the protein. Columns for positions covering the homorepeat itself were not included, as homorepeats diverge in length. To account for this, we included as additional variable the length of the homorepeat. As predictive variable we used the information provided by Interactome3D [16], as to whether a homorepeat overlaps with a region annotated to be involved in a protein-protein interaction.

The analysis was seeded and implemented in R with the caret package [19]. Data was randomly split in 70 % training and 30 % test. Training and test sets were checked to maintain the same proportion of positive and negative cases. To account for the imbalance of the classes (7.53 % positives in the polyQ dataset and 10.26 % for polyA), we downsampled the training data. We selected this method to deal with the unbalanced sets and avoid overfitting. The downsampling was performed by the “traincontrol” function from the caret package, through the “sampling = ‘down’” parameter. This function controls the training process of the model and, with this parameter, ensures that the number of samples from the majority class is reduced to match the number of samples in the minority class. By doing this, the potential bias in the training set due to class imbalance is mitigated, leading to more balanced and reliable model training.

Models were created with the following algorithms: random forest (RF), boosted logistic regression (logreg), k-nearest neighbors (knn), support vector machines with linear kernel (SVM), and neural network (NN). The optimized tuning parameter per model was mtry, nIter, k, C, and size and decay, respectively. The neural network model has a single hidden layer, which uses the logistic sigmoid activation function. The performance of each model was assessed from internal cross-validations (10-fold cross-validations repeated 3 times). Performance during the cross-validation is reported as precision, recall, F1 score, and area under ROC curve (AUC).

## 3 Results

### 3.1 Comparison of machine learning algorithms to predict protein interactions

Homorepeats polyQ and polyA have been described to have a functional role in protein-protein interactions (PPIs). However, not all of these regions participate in PPIs, and the linkage between polyX sequence and function has traditionally been done once an interaction is characterized. We hypothesized that the current knowledge of specific homorepeats annotated as interacting regions would allow a machine learning approach to identify which polyQ and polyA from the complete set of regions found in the human proteome are involved in PPIs.

**Fig. 1:**
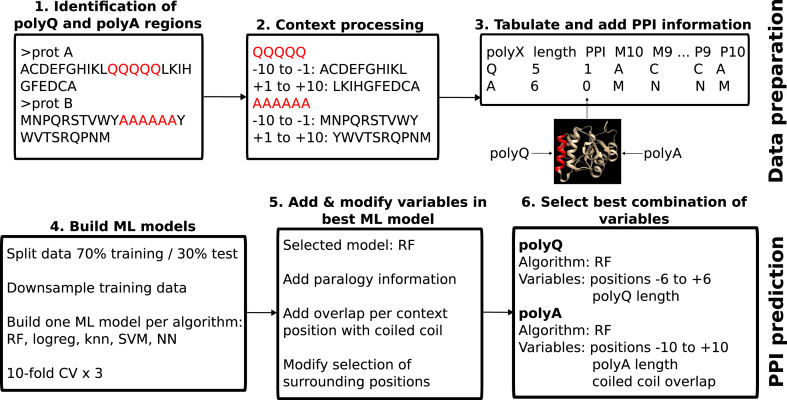
Flowchart illustrating data preparation, model construction and PPI prediction.

To this end, we first obtained the set of homorepeats in the human proteome, and then we calculated which of them are potentially involved in PPIs (“positives”) using as a proxy their overlap with regions known to be in interacting surfaces of a protein (see Methods for details). A total of 157 out of 2085 polyQ regions (7.53 % of the total), and 745 out of 7263 polyA regions (10.26 % of the total) were considered as involved in PPIs (Supplementary File 1; Supplementary File 2). Following previous works in which the amino acid context of homorepeats have proven of importance [[20], [21], [22]], we used the amino acids in the ten positions before and the ten positions after the homorepeat in the protein sequence to train several machine learning models (Fig. 1). The amino acid usage in these positions has been shown to be different with respect to the background proteome usage. Here we use these surrounding positions as an initial proxy for amino acids involved in the protein-protein interaction as well as the homorepeat; after the selection of the best machine learning model, we perform an optimization to find out which amino acid ranges are most useful for predictions (see section 3.3). The length of the homorepeat was also considered as a variable, as it has been shown to play a major role in the biology of these regions [23,24].

**Fig. 2:**
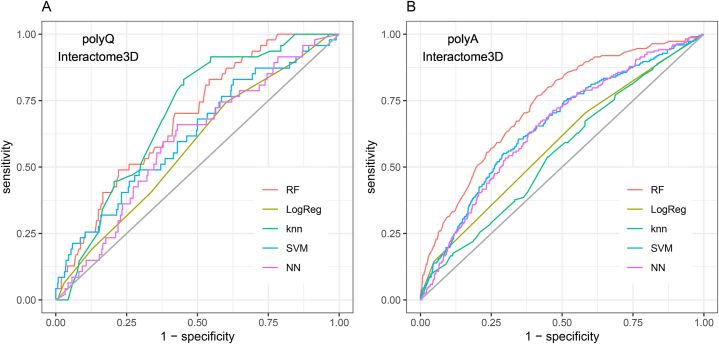
ROC curves obtained with machine learning models created with random forest (RF), boosted logistic regression (logreg), k-nearest neighbors (knn), support vector machines with linear kernel (SVM), and neural network (NN), for (A) polyQ and (B) polyA regions, with positive interaction data obtained from Interactome3D.

We compared the performance of the different machine learning models after applying them to the test dataset with parameters optimized in the training (Table 1; Fig. 2). Results are slightly better for models dealing with polyA regions than with polyQ regions. They all obtain high precision values (0.90–0.98) while recall values differ greatly (0.42–0.85).

**Table 1:** Performance of the best model obtained per algorithm in the test dataset, using data from Interactome3D.

	Algorithm	Parameter	Precision	Recall	F1	AUC	Accuracy
**PolyQ**	Random Forest	mtry = 200	0.9474	0.6851	0.7952	0.6861	0.6736
	Boosted Logistic Regression	nIter = 31	0.9320	0.6644	0.7758	0.5765	0.6448
	knn	k = 7	0.9764	0.5017	0.6629	0.6967	0.528
	SVM with linear kernel	C = 1	0.9433	0.5467	0.6922	0.6209	0.5504
	Neural Network	Size = 1, decay = 0.0001	0.9457	0.6332	0.7585	0.5979	0.6272
**PolyA**	Random Forest	mtry = 2	0.9414	0.7064	0.8071	0.7320	0.697
	Boosted Logistic Regression	nIter = 21	0.9253	0.4179	0.5758	0.5872	0.4472
	knn	k = 5	0.9026	0.8481	0.8745	0.5530	0.7815
	SVM with linear kernel	C = 1	0.9378	0.5550	0.6973	0.6551	0.5675
	Neural Network	Size = 1, decay = 0.01	0.9289	0.6619	0.7730	0.6499	0.6511

Based on a joint assessment of F1 score and area under the ROC curve (AUC) values, both for polyQ and polyA regions, the algorithm producing better results is random forest (RF). The AUC for the best RF model is 0.6861 for polyQ and 0.732 for polyA. These results were obtained by supplying the models with only the length of the homorepeats and the amino acids surrounding them acting as the context. Random forest (RF) outperformed other models due to its ability to capture complex, non-linear interactions between features, in this case the amino acids surrounding the homorepeats. By averaging multiple decision trees, RF reduces the risk of overfitting compared to single classifiers like logistic regression and SVM. The ensemble nature of RF benefits from the "wisdom of the crowd", leading to better generalization on unseen data, making it well-suited for our input dataset.

### 3.2 Paralogy information imputation

The set of homorepeats considered as positive for building the machine learning models in the previous approach is limited and restricted to data from Interactome3D. Paralogous proteins have similar sequences and, even if they interact with different proteins [25], they tend to use similar interfaces [26]. Here we explore the possibility to improve the models by adding polyQ- and polyA-containing paralogs of known interacting proteins as positives. To try to avoid false positives, the imputation of a homorepeat as positive is only done if the paralogous known interacting protein has an equivalent homorepeat annotated as positive, confirmed by the alignment of the paralogs.

The total number of positives improved slightly, from 157 to 179 out of 2085 for polyQ, and from 745 to 939 out of 7263 for polyA. The classes remain imbalanced; therefore, we applied the same methodology as detailed before. Results do not differ from the ones obtained without paralogy information (Table 2; Supplementary Fig. 1), with similar ranges for precision (0.89–0.96) and recall values (0.36–0.85).

**Table 2:** Performance of the best model obtained per algorithm in the test dataset, using data from Interactome3D and paralogy information.

	Algorithm	Parameter	Precision	Recall	F1	AUC	Accuracy
**PolyQ**	Random Forest	mtry = 200	0.9456	0.6392	0.7628	0.6646	0.6362
	Boosted Logistic Regression	nIter = 21	0.9444	0.4764	0.6333	0.6130	0.4952
	knn	k = 9	0.9601	0.4641	0.6257	0.6928	0.492
	SVM with linear kernel	C = 1	0.9351	0.6060	0.7354	0.6475	0.601
	Neural Network	Size = 1, decay = 0.1	0.9261	0.5709	0.7064	0.5881	0.5657
**PolyA**	Random Forest	mtry = 2	0.9228	0.7433	0.8234	0.7241	0.7222
	Boosted Logistic Regression	nIter = 31	0.9173	0.3569	0.5139	0.5857	0.4118
	knn	k = 5	0.8914	0.8477	0.8690	0.6162	0.7773
	SVM with linear kernel	C = 1	0.8989	0.6753	0.7712	0.6183	0.6511
	Neural Network	Size = 1, decay = 0.1	0.9003	0.7043	0.7903	0.6423	0.6745

The expected added value by introducing paralog information to the pool of positive homorepeats does not generally translate into better predictions. Performance improves only for RF in some metrics when using paralogy, with an F1 score of 0.8234 and 0.8071, accuracy of 0.7222 and 0.6970, but AUC values of 0.7241 and 0.7320, for models built with and without paralogy information, respectively. In order to keep the models as simple as possible, and given the slight improvement, if any, in the prediction performance of the models, we decided not to use paralogy information going forward.

Up to this point, the algorithm for which we obtain better predictions (AUC polyQ = 0.6861, AUC polyA = 0.732) is random forest, with values of 200 for polyQ and 2 for polyA for the parameter mtry (optimal number of variables to randomly sample as candidates per split), and using as variables all context positions from −10 to +10 and the homorepeat length.

### 3.3 Model optimization by context selection and coiled coil information

Next, we studied the effect of decreasing the size of the surrounding regions for the amino acid context. The results improve for polyQ regions when building the random forest algorithm only with amino acid positions from −6 to +6 (Supplementary Table 1; Supplementary Fig. 2A), raising the AUC value from 0.6861 to 0.7154. For polyA regions, there was no improvement, validating our initial selection as features of the ten preceding and the ten following amino acids (Supplementary Fig. 2B).

Since polyQ is known to follow coiled coils [3] and this has been proposed as a hallmark of interacting polyQ [27], we next considered proximity to coiled coils as a feature both for polyQ and polyA. We built one additional machine learning model per homorepeat type, with their respective best current conditions (random forest and positions −6 to +6 for polyQ, and random forest and positions −10 to +10 for polyA), and adding as variables the overlap of each context position with a coiled coil region. The total number of machine learning variables used to build the new model for polyQ was 25 (12 context positions, 12 overlaps of each position with coiled coil, plus the polyQ length), and 41 variables for polyA (20 context positions, 20 overlaps of each position with coiled coil, plus the polyQ length).

The results of the model for polyQ are not better with the coiled coil information (precision = 0.9515, recall = 0.6782, F1 = 0.7919, AUC = 0.6752; Supplementary Fig. 3A). However, there is an improvement for polyA (precision = 0.9477, recall = 0.7141, F1 = 0.8145, AUC = 0.7451; Supplementary Fig. 3B).

### 3.4 Applying the best machine learning models to generate predictions

We have trained, tested and optimized two machine learning models to independently predict which polyQ and polyA regions are involved in protein-protein interactions. Here we use these models to do the actual predictions on the complete sets of polyQ and polyA (Supplementary File 3; Supplementary File 4). For polyQ, it is a random forest model using as variables the amino acids in positions from −6 to +6, and the polyQ length. For polyA, it is a random forest model using as variables the amino acids in positions from −10 to +10, the polyA length, and the overlap of each of the positions with a coiled coil region (Supplementary Fig. 4).

**Fig. 3:**
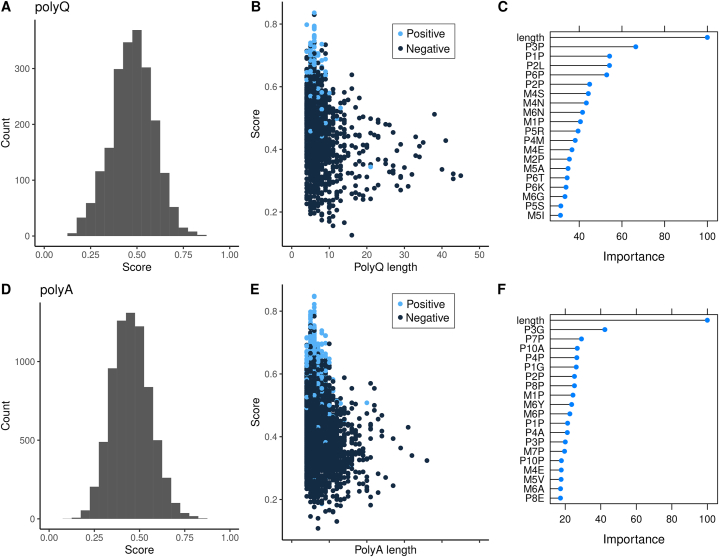
**(A)** Distribution of prediction scores for polyQ regions. **(B)** Distribution of predictions scores versus the polyQ length, for polyQ regions known to interact (positives) and unknown (negatives). **(C)** Importance of top20 machine learning variables for the prediction for polyQ regions; the first character denotes if it is a plus (P) or a minus (M) position, the second the position, and the third the amino acid. **(D)** Distribution of prediction scores for polyA regions. **(E)** Distribution of predictions scores versus the polyA length, for polyA regions known to interact (positives) and unknown (negatives). **(F)** Importance of top20 machine learning variables for the prediction for polyA regions; the first character denotes if it is a plus (P) or a minus (M) position, the second the position, and the third the amino acid.

The prediction score represents the probability of a given homorepeat to interact with another protein, as predicted by the best models developed. The score distributions for polyQ and polyA are very similar (Fig. 3A and **D**). Homorepeats experimentally determined to interact (positives) obtain higher scores than those for which no information is available (Fig. 3B and **E**), as expected. In the negative dataset, there are 13 polyQ and 12 polyA regions with a score greater than 0.7 (Supplementary File 3; Supplementary File 4), meaning that it is highly probable that they do interact with other proteins.

We calculated the importance of each variable to build the models, with values in the range from 0 to 100 (Fig. 3C and **F**). For both polyQ and polyA the most important feature is the homorepeat length. The length distribution of the positives is more skewed towards short homorepeats, which translates to it being of major importance for the predictions. Interestingly, the second most important feature is somehow shared between polyQ and polyA, which is having proline or glycine in the third position after the polyQ (P3P) and polyA (P3G), respectively. These results are in line with previous observations of an enrichment of prolines and glutamines C-terminally to polyQ and polyA regions [22,24,28].

### 3.5 Evaluation of selected predictions

In this section, we illustrate the value of the predictions with a few novel predictions with extremely high (interacts) or low (does not interact) scores. These cases can be found in Supplementary Files S3 and S4 for polyQ and for polyA, respectively. For regions predicted to interact, we examined structures in the PDB of homologs looking for an aligned region involved in an interaction, following the expectation that homologs have similar interfaces for interaction.

#### 3.5.1 Interacting polyQ in ubiquitin carboxyl-terminal hydrolase 6

**Fig. 4:**
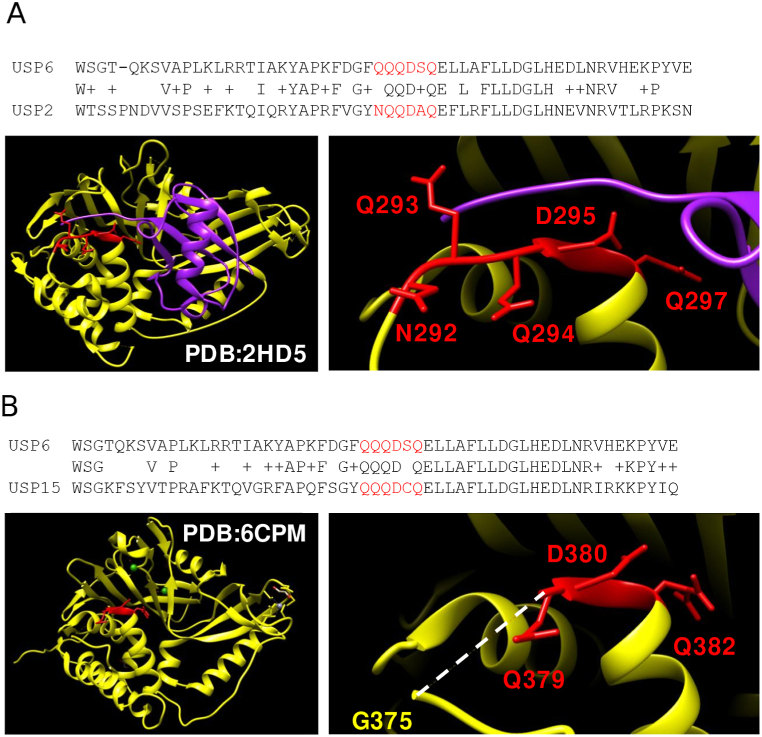
**(A)** Pairwise alignment of human Ubiquitin carboxyl-terminal hydrolase 6 (USP6) with Ubiquitin carboxyl-terminal hydrolase 2 (USP2). Structure of human USP2 (yellow) with ubiquitin (purple) (PDB:2HD5). The polyQ region is marked in red and focused in the inset. Key amino acids are indicated. **(B)** Pairwise alignment of human Ubiquitin carboxyl-terminal hydrolase 6 (USP6) with Ubiquitin carboxyl-terminal hydrolase 15 (USP15). Structure of human USP15 (yellow) (PDB:6CPM). The polyQ region is marked in red and focused in the inset. Key amino acids are indicated. Missing signal for fragment 376YQQ378 (discontinuous line) suggests that it is flexible.

For polyQ, the prediction of interaction with the highest interaction score (0.830) and no experimental evidence is the second best: it is for a polyQ in positions 624–638 for human Ubiquitin carboxyl-terminal hydrolase 6 (UniProtKB:P35125) with sequence “QQQDSQ”, a 4/6 polyQ. This is predicted to be part of a Ubiquitin specific protease (USP) domain (InterPro:IPR028889) running from positions 532 to 1369. This domain is present in other Ubiquitin carboxyl-terminal hydrolases. The region is Q-rich in other human ubiquitin carboxyl-terminal hydrolases (Fig. 4). No other polyQ was identified for UBP6.

The structure of the homolog human Ubiquitin carboxyl-terminal hydrolase 2 (USP2) was solved in complex with ubiquitin (PDB:2HD5; [29]). The Q-rich region interacts with the N-terminal of ubiquitin (Fig. 4A).

We note that in other USP structures where the region is free, the N-terminal part is unresolved, indicating that it is disordered, whereas the end that forms the start of the next helix appears ordered. We show an example for USP15 (Fig. 4B) (PDB:6CPM; [30]). We hypothesize that the Q enrichment gives the region flexibility and that upon interaction the region is fixed in a coil structure next to the following helix.

The motif QQD is functionally important and conserved in these three sequences and it is identified as QQD box in early analyses of USPs, which reported that the D is absolutely conserved in the family [31].

#### 3.5.2 Non-interacting polyQ in nuclear receptor coactivator 6

The polyQ prediction with the lowest interaction score (0.126) is for sequence “QPPQQQPQPQLPQQQQ” in positions 999–1014 of the Nuclear receptor coactivator 6 (NCOA6; UniProtKB:Q14686), a large protein with a large content of disorder (97 % according to MobiDB [32]). In fact, the protein has a total of eight polyQ (highest score 0.470) but they are all in disordered regions. The 2063 amino acid protein has just two 30 amino acid segments situated at 60 and 100 amino acids from the N-terminal, and the closest polyQ is more than 100 amino acids apart from those. This region is annotated as InterPro:IPR032715 Nuclear receptor coactivator 6, TRADD-N domain (positions 47–190) with distant homology to several bacteria [33].

This protein is likely interacting via linear motifs but to this day there is no structure in the PDB for this protein or fragments, alone or in complexes. For example, one of the best characterized interactions according to the HIPPIE database [34] is with retinoid X receptor alpha (RXRA; UniProtKB:P19793). This is using an LXXLL motif (present in human NCOA6 from 887 to 891), which is used by many coactivator molecules to bind ligand binding domains of nuclear receptors; in this interaction between NCOA6 and RXRA, nearby S884 makes the interaction more specific [35]. No polyQ was at less than 100 amino acids from this motif.

#### 3.5.3 Interacting polyA in Heterogeneous nuclear ribonucleoprotein C-like 3

For polyA, the prediction with the highest interaction score (0.784) and no experimental evidence is the thirteenth best: it is for a polyA in positions 63–68 for Heterogeneous nuclear ribonucleoprotein C-like 3 (HNRNPCL3; UniProtKB:B7ZW38) with sequence “ARAAVA”. Similarly to the top polyQ example shown above, this polyA was short and impure (4 of 6 A residues) and also was the only polyA identified in the protein. The polyA is part of a RRM domain (RNA recognition motif domain; InterPro:IPR000504) running from positions 16 to 87. This domain is one of the most ubiquitous single stranded RNA binding domains [36]. It is composed of a beta sheet of four beta strands and two helices with notation and order beta1-alphaA-beta2-beta3-alphaB-beta4. In HNRNPCL3, the A-rich region is in the second helix (alphaB) that runs from positions 60 to 70 with sequence “EKNARAAVAGE”.

The beta sheet binds the RNA on one side while the two helices pack to the other side allowing interactions with proteins that do not potentially interfere with the RNA recognition [36].

**Fig. 5:**
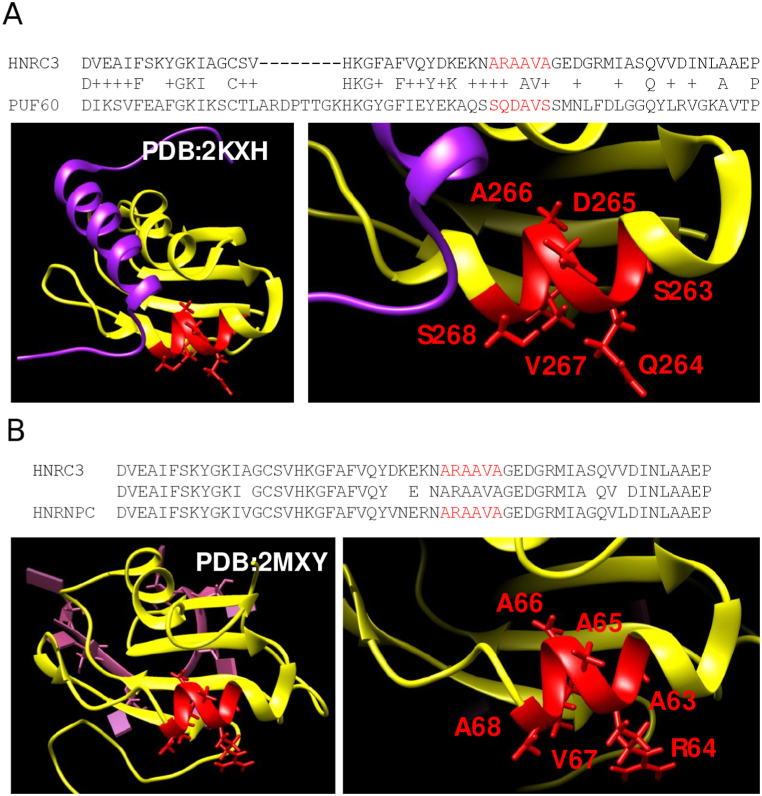
**(A)** Pairwise alignment of human Heterogeneous nuclear ribonucleoprotein C-like 3 (HNRC3) with Poly(U)-binding-splicing factor (PUF60; a.k.a. FIR). Structure of human FIR's (yellow) RRM2 domain in complex with the Nbox peptide from FBP (purple) (PDB:2KXH). The polyA region is marked in red and focused in the inset. Key amino acids are indicated. **(B)** Pairwise alignment of human Heterogeneous nuclear ribonucleoprotein C-like 3 (HNRC3) with 91 % identical Heterogeneous nuclear ribonucleoproteins C1/C2 (HNRNPC). Structure of the protein (yellow) in complex with 5′-AUUUUUC-3′ RNA (pink) (PDB:2MXY). The polyA region is marked in red and focused in the inset. Key amino acids are indicated.

There is a structure for the interaction of the two RRM1 and RRM2 domains (positions 103 to 297) from the Poly(U)-binding-splicing factor PUF60 (FUSE-binding protein-interacting repressor, FIR) with a fragment (Nbox peptide, positions 27 to 52) from Far upstream element-binding protein 1, FUBP1 (FBP; PDB:2KXH; [37]). FIR (PUF60) is a DNA-binding protein, which has two RRM domains in tandem, RRM1 and RRM2, which bind to FBP. The RRM1 is known to bind DNA [38] but the structure of the tandem RRM1-RRM2 suggests that the packing of RRM2 against RRM1 makes it impossible to use its beta-sheet for DNA binding [37]. In this structure, RRM2 interacts with a fragment from FBP (FUBP1) that adopts a helical structure using the helix alphaA in anti-parallel orientation, and with helix alphaB. The C-terminal part that aligns with the polyA in HNRNPCL3 (sequence “SQDAVS”) contacts the N-terminal of the FBP peptide (Fig. 5A). We note that, like the previous example, this region contains a central “QDA” motif, but the inter-molecular contacts and structure are different. The structure of the two same tandem repeats from FIR in a dimer bound to the FUSE DNA without the peptide (PDB:2QFJ; [38]) does not show differences in the extent of the A-rich helixB (not shown).

However, the situation is different if we examine the polyA in the structure of a closer homolog with the same polyA than HNRNPCL3. The structure of Heterogeneous nuclear ribonucleoproteins C1/C2 (HNRNPC; 91 % identical to HNRNPCL3) in complex with 5′-AUUUUUC-3′ RNA is available (PDB:2MXY; Fig. 5B; [39]). The helix ends at the polyA as it is three residues shorter than in the FIR structures. If this region binds a protein, as we predict, it would be interesting to see if this helix would be extended C-terminally as in FIR.

#### 3.5.4 Non-interacting polyA in Ran-binding protein 9

The polyA prediction with the lowest interaction score (0.108) is for sequence “ASAAAPA” in positions 93–99 of the Ran-binding protein 9 (RANBP9; UniProtKB:Q96S59). This protein has 729 amino acids and a low content of disorder (5 % according to MobiDB), but the intrinsically disordered region (IDR) occupies the 150 N-terminal and includes the polyA. The proteins contains another polyA, which happens in the same IDR (positions 62 to 66). This is a pure polyA and has also a low interaction score (0.316). The IDR is very compositionally biased and contains other seven homorepeats (5 polyP, a polyQ, and a polyG).

These examples suggest that while polyA and polyQ can be found in disordered regions, at least based on the training data, their function is not interacting as, in such an environment, they do not have the capacity to form the alpha helical structure that contributes a globular surface for interaction.

### 3.6 A conflicting prediction: an interacting polyQ predicted not to interact

To further examine the performance of the method, we were curious to examine conflicting predictions of a polyQ used as positive interactor in the training data, with the worst interaction scores.

The case of the N-terminal of polyQ in Huntingtin (sequence 18 to 38; UniProtKB:P42858) is the experimental positive with the lowest score (0.344). This region is well-known, as its genetic expansion results in neurodegenerative disease and modifies the interactions of the protein (see e.g. Ref. [40]). Examination of the current experimental evidence is only based on Huntingtin fragments and constructs of abnormally expanded polyQ and reports self-interactions. There is currently no known structure of a complex of native Huntingtin with the polyQ region.

**Fig. 6:**
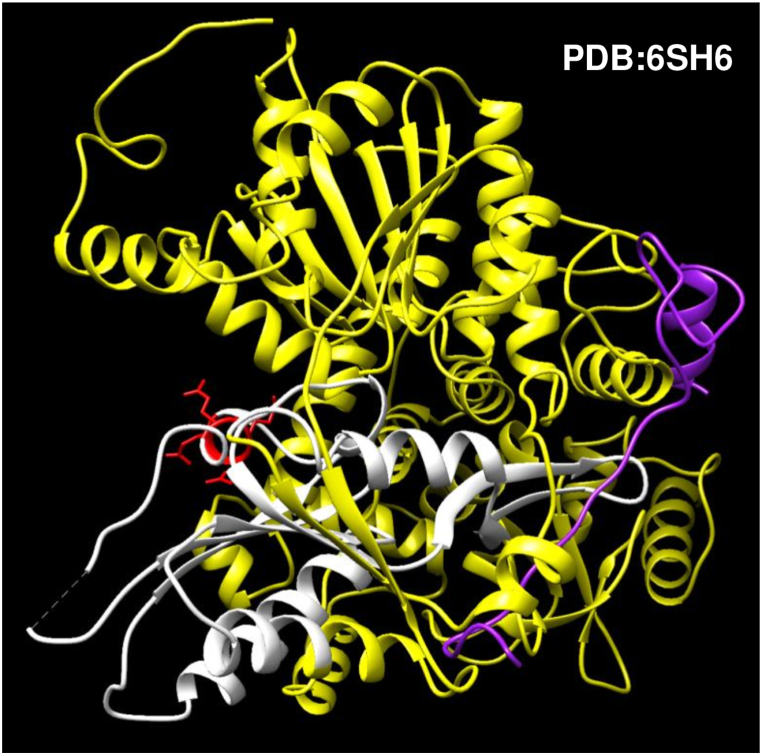
Structure of the ATP-dependent RNA helicase DHX15 in complex with a fragment from NKRF containing a G-patch motif (purple). A domain in DHX15 (positions 338 to 476) is marked in white (with the polyQ in red) and the rest of the sequence in yellow. The polyQ is in the side of DHX15 opposite the interacting molecule.

The next case is a short pure polyQ (positions 395 to 398) in the ATP-dependent RNA helicase DHX15 (UniProtKB:O43143), which receives a similar low interaction score of 0.394. The database indicates based on PDB:6SH6 that the fragment 338–476 interacts with a fragment (positions 553 to 593) containing the G-patch motif of the NF-kappa-B-repressing factor (NKRF; a.k.a. GPANK1; UniProtKB:O95872; [41]). However, this amino acid range defines a domain, which includes the polyQ, with many residues (the polyQ included) not interacting (Fig. 6). We assume that this is an annotation error that might be corrected in the future.

## 4 Discussion

Our study compares various machine learning algorithms to predict protein-protein interactions (PPIs) involving polyQ and polyA. These homorepeats have a functional role in protein-protein interactions. However, not all participate in PPIs, and the involvement of such polyX sequences in interactions is typically established after the interaction is characterized experimentally, for example by solving the structure of the protein complex. We hypothesized that utilizing current knowledge of specific homorepeats annotated as interacting regions could facilitate a machine learning approach to identify which polyQ and polyA regions from the entire set in the human proteome are involved in PPIs.

We initially obtained the set of homorepeats in the human proteome and identified those potentially involved in PPIs based on their overlap with known interacting surfaces of proteins. Machine learning models were then trained using the amino acids surrounding the homorepeats and the length of the homorepeat as variables. The performance of various machine learning algorithms was evaluated, with random forest (RF) demonstrating superior performance in predicting interactions involving both polyQ and polyA regions. Performance evaluation of the algorithms used standard metrics such as precision, recall, F1 score, and area under the ROC curve (AUC). The results showed that RF models exhibited robust performance in predicting PPI involvement for both polyQ and polyA regions, achieving AUC values of 0.6861 and 0.732, respectively. Because paralogs tend to use similar interfaces to interact with proteins, we then annotated polyX aligning with interacting polyX in paralogs and added them to the set of positives. But this did not significantly enhance model performance and the idea was discarded.

Further refinement of the models involved the selection of informative amino acid positions surrounding the homorepeat. This optimization led to improved predictions for polyQ regions, whereas the original feature set remained most effective for polyA predictions. Subsequent application of the optimized machine learning models to the complete sets of polyQ and polyA regions in the human proteome generated probability scores indicating the likelihood of each homorepeat being involved in PPIs.

The importance of each variable in the models was also assessed, with homorepeat length identified as the most crucial feature for both polyQ and polyA predictions. However, a few differences were observed for polyQ and polyA. Overlap to CC regions helped polyA predictions but not polyQ, although polyQ has been noted for following CC. PolyQ predictions were improved when using only the six surrounding amino acids, while for polyA ten amino acids were better. Taken together, these results suggest that polyA requires a wider sequence environment to participate in interactions compared to polyQ.

To further test our model's performance, we wondered if our annotated sets of polyX aligning with interacting polyX in paralogs received scores significantly higher than negatives. For polyQ, the average score of the paralog validated regions was 0.54, which was lower than the average for the positives (0.62), but significantly higher than the average for the negatives (0.46; p-value = 0.01, Wilcoxon rank sum test with continuity correction). For polyA, the average score of the paralog validated regions was 0.49, lower than the average for the positives (0.59), but significantly higher than the average for the negatives (0.44; p-value = 9.9e-07, Wilcoxon rank sum test with continuity correction). These results suggest that polyQ and polyA share properties across paralogs regarding their ability to participate in PPIs.

For the cases with the highest interaction prediction scores and no experimental information, we were able to find homologs with aligned interacting regions by examination of structures of complexes in the PDB. Conversely, the experimental information supporting the interactions of the regions receiving the lowest scores were dubious on close inspection.

In conclusion, we demonstrated the potential of machine learning algorithms in predicting PPI involvement of homorepeat regions within the human proteome.

The models provide a number of sequence positions and amino acids relative to the homorepeat, which could be used to direct mutational studies exploring the participation of polyQ and polyA in interactions. In addition, our tables scoring all polyQ and polyA, particularly the prediction of those to be involved in interactions, could be used to guide studies of protein interactions.

We note that many of the identified homorepeats predicted to be associated with PPIs are Q/A-rich regions with other interspersed residues (e.g. “QQQDSQ”, “ASAAAPA”) and not pure polyQ or polyA tracts. The extension of the latter is responsible for the pathogenesis of many neurodegenerative diseases. We take this as an indication that the homorepeats we identified are probably not prone to aggregation and are not related to this type of diseases.

The method has been trained with a relatively limited number of positives. We do not know how the experimental limitations in obtaining structural and other type of information regarding protein interactions might have affected the breadth of available experimentally proven interactions. This is particularly important in the case of sequences like polyQ and polyA which, as many compositionally biased sequences do, can be difficult to handle in experiments. For example, the polyQ in Huntingtin could be receiving a low score by our predictor because it could be using a mode of interaction that cannot be resolved with the methods used for our set of positives. In this respect, it is important to note that while we predict the involvement of certain polyQ and polyA in PPIs, this does not mean that other polyQ and polyA do not participate in PPIs: the future expansion of interaction and structural data and the possibility of integrating other data types could one day provide new features that we could use to demonstrate the involvement of further polyQ and polyA in PPIs.

While our work focused on human proteins, it will be possible to extend our approach to other species. However, while human proteins have a relatively high degree of interaction and structural experimental data, working with other species will be challenging as the amount of experimental data decreases and will require evolutionary comparisons.

Despite some limitations, our approach offers valuable insights into the functional roles of polyQ and polyA regions. It provides a framework that could integrate novel information about specific interactions of polyQ and polyA, which will allow further refinement and validation of the models, enhancing their reliability and applicability in biological research.

## Ethics approval and consent to participate

Not applicable.

## Consent for publication

Not applicable.

## Availability of data and materials

The codes developed and the datasets used and/or analyzed are available under a GPL-3.0 license from the following dedicated github repository: https://github.com/pmiemun/polyX_ML.

## Funding

The authors have received no specific funding for this work.

## CRediT authorship contribution statement

**Pablo Mier:** Conceptualization, Data curation, Formal analysis, Investigation, Methodology, Software, Validation, Writing – original draft, Writing – review & editing. **Miguel A. Andrade-Navarro:** Data curation, Formal analysis, Investigation, Supervision, Validation, Writing – original draft, Writing – review & editing.

## Declaration of competing interest

The authors declare that they have no competing interests.
