# Supplementary material for: Predicting the involvement of polyQ- and polyA in protein-protein interactions by their amino acid context
Source: Heliyon. 2024 Sep 14;10(18):e37861. doi: 10.1016/j.heliyon.2024.e37861 (PMC11422028; doi:10.1016/j.heliyon.2024.e37861)
Supplement: Multimedia component 1 [file mmc1.docx]

**Supplementary Table 1**. Performance in the test dataset of random forest models build with different combinations of amino acid positions surrounding the homorepeats.

|  | **Context positions** | **Precision** | **Recall** | **F1** | **AUC** |
| --- | --- | --- | --- | --- | --- |
| **PolyQ** | -10 → +10 | 0.9474 | 0.6851 | 0.7952 | 0.6861 |
|  | -9 → +9 | 0.9461 | 0.6678 | 0.7830 | 0.6851 |
|  | -8 → +8 | 0.9576 | 0.6246 | 0.7560 | 0.6812 |
|  | -7 → +7 | 0.9525 | 0.6592 | 0.7791 | 0.6848 |
|  | -6 → +6 | 0.9637 | 0.5969 | 0.7372 | 0.7154 |
|  | -5 → +5 | 0.9489 | 0.6747 | 0.7887 | 0.6650 |
|  | -4 → +4 | 0.9592 | 0.6107 | 0.7463 | 0.7022 |
|  | -3 → +3 | 0.9468 | 0.6471 | 0.7688 | 0.6941 |
|  | -2 → +2 | 0.9467 | 0.6142 | 0.7450 | 0.6724 |
|  | -1 → +1 | 0.9466 | 0.5830 | 0.7216 | 0.6663 |
| **PolyA** | -10 → +10 | 0.9414 | 0.7064 | 0.8071 | 0.7320 |
|  | -9 → +9 | 0.9390 | 0.6767 | 0.7866 | 0.7165 |
|  | -8 → +8 | 0.9401 | 0.6660 | 0.7796 | 0.7105 |
|  | -7 → +7 | 0.9412 | 0.6793 | 0.7891 | 0.7026 |
|  | -6 → +6 | 0.9330 | 0.6834 | 0.7889 | 0.6966 |
|  | -5 → +5 | 0.9339 | 0.6430 | 0.7616 | 0.6833 |
|  | -4 → +4 | 0.9337 | 0.6343 | 0.7554 | 0.6754 |
|  | -3 → +3 | 0.9327 | 0.5811 | 0.7160 | 0.6592 |
|  | -2 → +2 | 0.9390 | 0.5586 | 0.7004 | 0.6681 |
|  | -1 → +1 | 0.9373 | 0.4813 | 0.6360 | 0.6261 |
