# Supplementary figures and images for: Predicting the involvement of polyQ- and polyA in protein-protein interactions by their amino acid context
Source: Heliyon. 2024 Sep 14;10(18):e37861. doi: 10.1016/j.heliyon.2024.e37861 (PMC11422028; doi:10.1016/j.heliyon.2024.e37861)

A

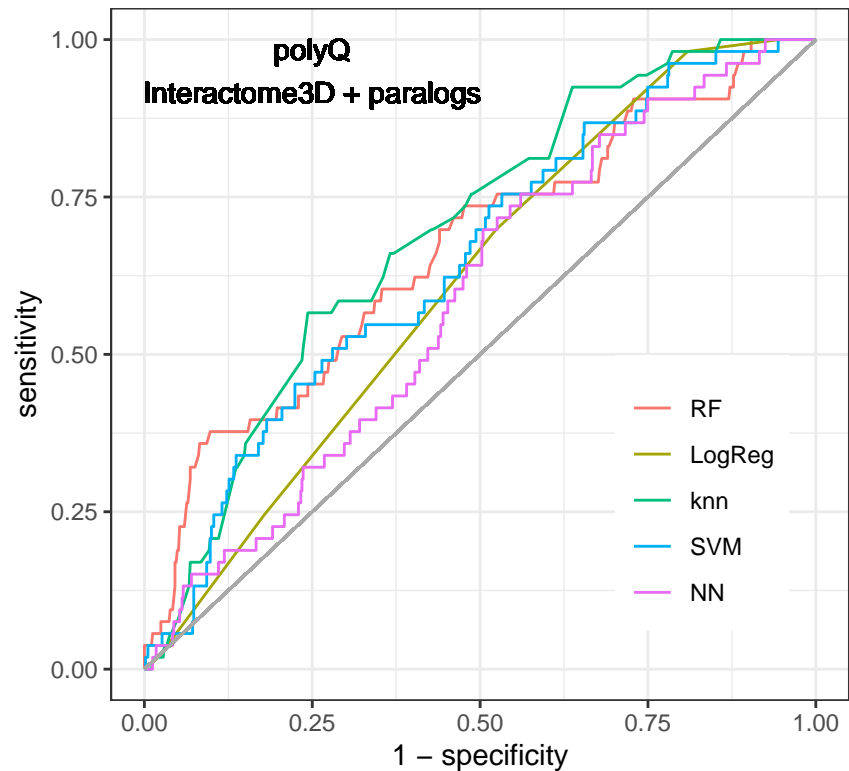

B

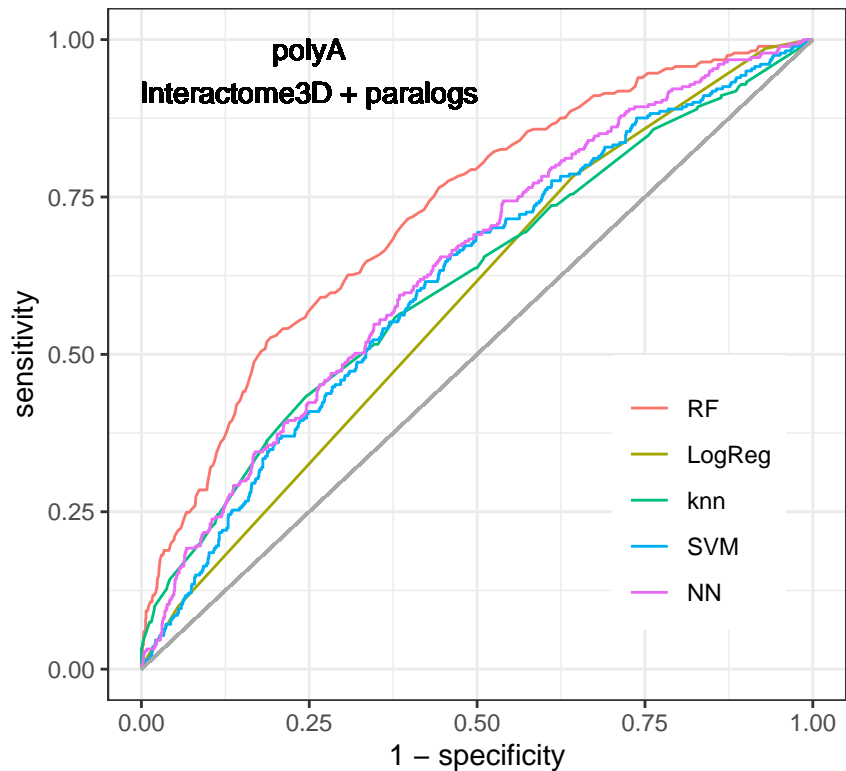

Supplement: Multimedia component 2 [file mmc2.pdf]

A

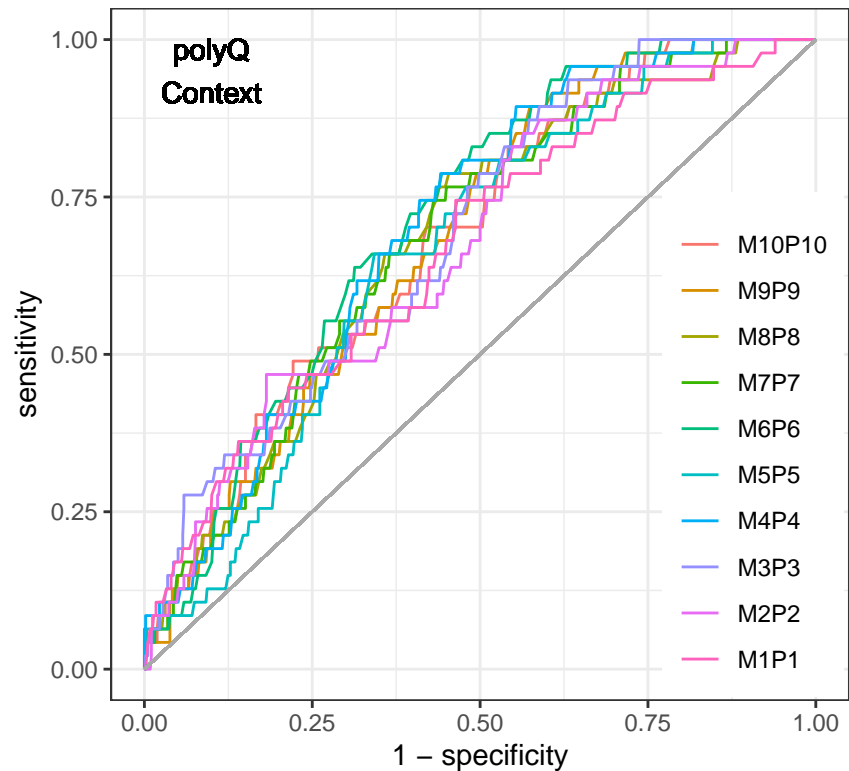

B

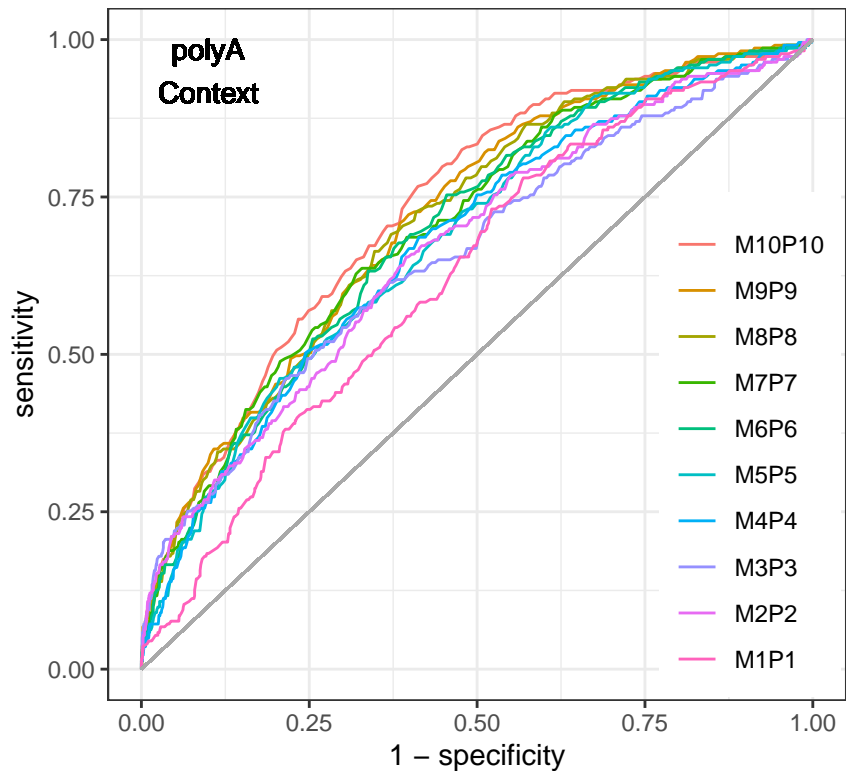

Supplement: Multimedia component 3 [file mmc3.pdf]

A

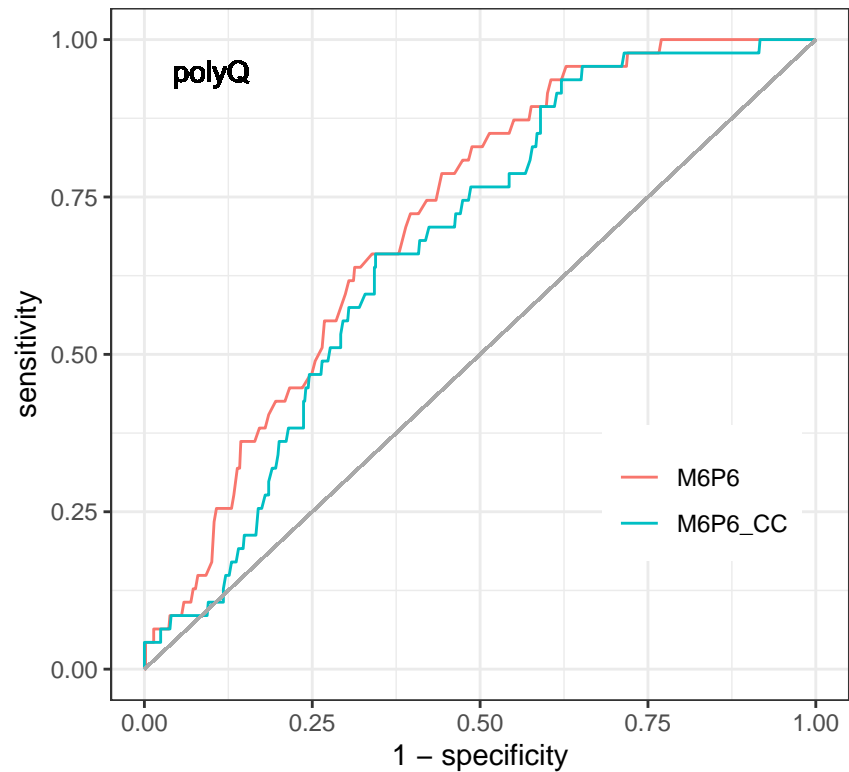

B

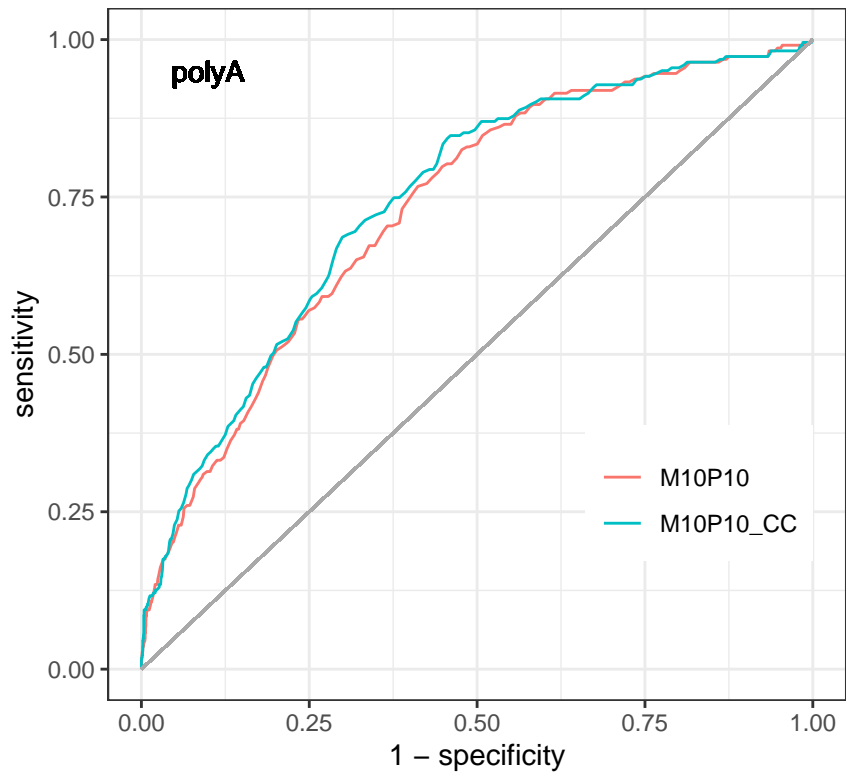

Supplement: Multimedia component 4 [file mmc4.pdf]

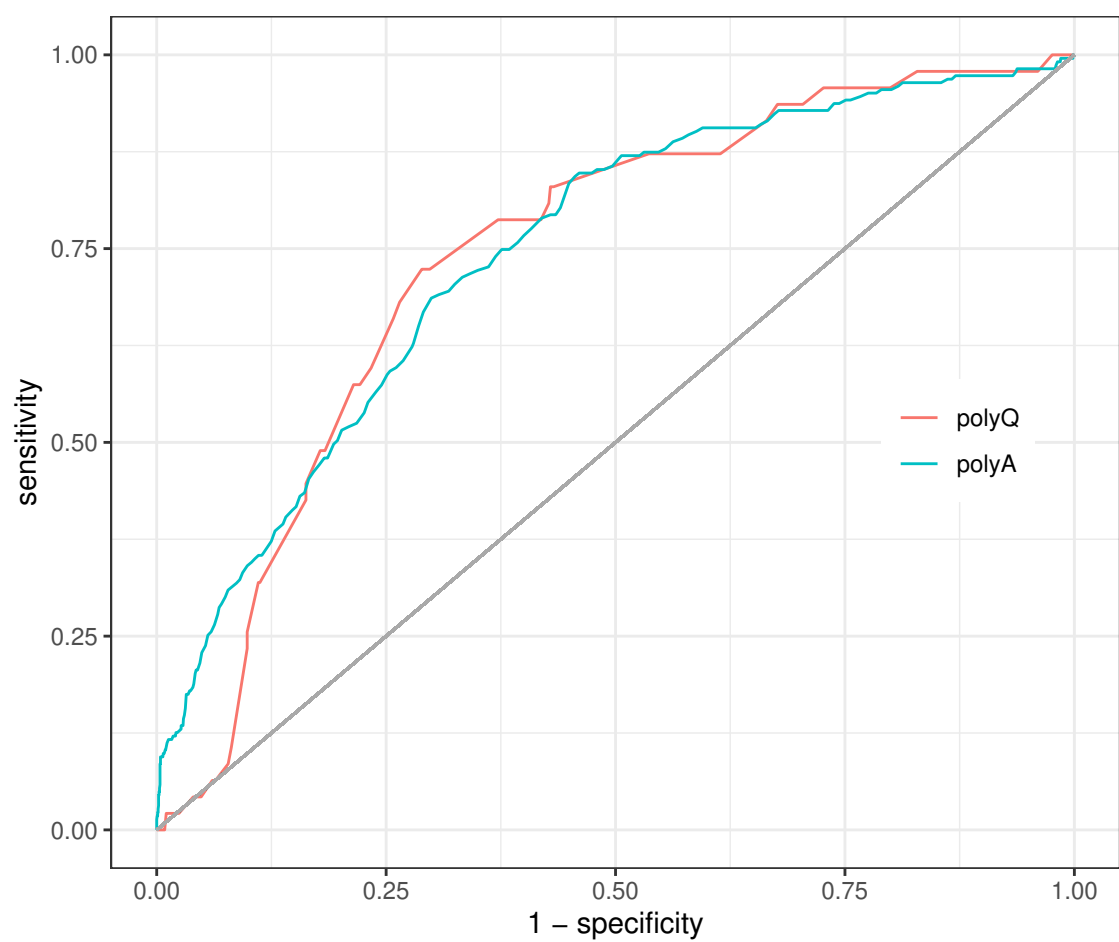

Supplement: Multimedia component 5 [file mmc5.pdf]
